# From Lab to Limb: Unraveling Translational Insights and Significance of Animal Models in Lower Extremity Transplantation

**DOI:** 10.1016/j.jpra.2024.11.018

**Published:** 2024-11-28

**Authors:** Yalcin Kulahci, Naga Anvesh Kodali, Zeynep Demir, Omer Dirican, Bedreddin Sazoglu, Ramu Janarthanan, Fatih Zor, Vijay S. Gorantla

**Affiliations:** a Department of Surgery, Wake Forest School of Medicine, Winston Salem, North Carolina, USA; b Department of Plastic and Reconstructive Surgery, Amrita Institute of Medical Sciences and Research Center, Amrita Vishwa Vidyapeetham, Kochi, India; c Department of Plastic Surgery, Indiana University, Indianapolis, Indiana, USA

**Keywords:** Lower extremity transplantation, LET, Vascularized composite allotransplantation, VCA, Animal models

## Abstract

The advancements in medicine throughout the twentieth century have been largely attributed to animal studies. The initial step in researching an animal disease is to establish a model closely resembling the clinical circumstances in humans. Consequently, an excellent animal model is essential for almost any experimental research. The aim of this review is to evaluate the current research on animal models for lower extremity transplantation (LET) and determine how pertinent and significant these models are for therapeutic settings. To bring the reader up to date from an allotransplantation standpoint, we also review, assess, and highlight the noteworthy and intriguing results of the clinical cases performed so far and various animal models. The discussion of their clinical applicability and practicality in the present and future has shed light on the experience with vascularized composite allotransplantation (VCA) around the globe.

## Introduction

The first successful hand transplant was performed in 1998, marking the start of a new era in clinical reconstructive transplantation almost 2 decades ago.^1^ The first face transplant took place in 2005,^2^ and since then, more vascularized composite allotransplants have been used in clinical settings. Lower Extremity Transplantation (LET) represents one of the most intriguing and latest attempts at vascularized composite allotransplantation (VCA). So far, 5 cases of LET^3^, ^4^, ^5^, ^6^, ^7^, ^8^, ^9^, ^10^, ^11^, ^12^, ^13^, ^14^ have been performed worldwide. Nonetheless, this process still sparks debates in the scientific community regarding medical and ethical challenges.^3^^,^^15^, ^16^, ^17^ The purpose of this study is to review the relevant reported animal models^9^^,^^18^, ^19^, ^20^, ^21^, ^22^, ^23^, ^24^, ^25^, ^26^, ^27^, ^28^, ^29^, ^30^, ^31^, ^32^, ^33^, ^34^, ^35^, ^36^, ^37^, ^38^, ^39^, ^40^, ^41^ of LET and discuss how well they replicate human applications, as well as their applicability in clinical settings.

## Brief Review of the Clinical Cases of LET

Till date, 5 cases of LET have been reported. The first successful complete LET was performed between ischiopagus twins by Zuker et al. in 2006.^8^^,^^9^^,^^13^^,^^14^^,^^42^ They transplanted a functioning limb from the twin who was not expected to survive due to a lethal cardiac anomaly. The limb survived, and they also published their 6-year follow-up.^8^ Despite these encouraging results, it was not suggested as a “real allotransplantation case” or “real VCA,” as immune suppressive therapy was not required because of immune compatibility between twins. Additionally, as the patient was a newborn, the distance needed for nerve regeneration was not too extensive.

In 2011, Cavadas et al. performed the world's first bilateral transfemoral allotransplantation case of lower extremities. The recipient was a 22-year-old male, and the donor was a full HLA-mismatched female multiorgan donor.^7^^,^^43^ During the long-term follow-up at the 15th postoperative month, primary central nervous system posttransplant lymphoproliferative disease developed. Consequently, the immunosuppressive protocol was immediately discontinued, and both allografts were removed.^43^

The world's second LET concurrent with bilateral upper extremity (in other words, the first triple extremity transplantation) was performed in 2012 in Turkey by Ozkan et al. Despite the absence of publication in the literature, data from this case have been presented at scientific meetings.^9^^,^^13^ Unfortunately, on the first postoperative day, the lower extremity allograft had to be removed due to perioperative hemodynamic problems. Five months after allotransplantation, disseminated aspergillosis and renal failure developed, leading to the patient's death.^9^

In 2012, Nasir et al. performed the first quadruple extremity transplantation in Turkey.^9^, ^10^, ^11^, ^12^, ^13^ Unfortunately, the patient died on the 4th day following the allotransplantation due to perioperative hemodynamic complications.

In 2017, Arudchelvam et al. performed the world's fifth and most recent LET in Sri Lanka.^3^^,^^4^^,^^42^ The recipient, a 32-year-old male with an above-knee amputation, received the transplant from a 52-year-old male donor with compatible HLA and lymphocyte cross match. Six months post-transplantation, the patient exhibited movement in his knee joint and could bear some weight. Additionally, he reported a burning sensation at the ankle joint level, indicating early signs of sensory recovery. Personal communication with the author^44^ revealed that the patient has been ambulating with incomplete motor and sensory recovery. Dr. Arudchelvam further disclosed that during the 6-year follow-up period, the patient experienced frequent episodes of lymphangitis and lymphedema in the transplanted lower extremity. These episodes were attributed to the patient's noncompliance with using compression stockings for the control of lymphedema.

As mentioned earlier, clinical experience in LET is not only limited but also yields very severe outcomes. One of the reasons for these discouraging results is the lack of suitable animal models, which could aid in understanding the perioperative and long-term challenges of LET. In this review, we summarize the animal models of LET and discuss their clinical relevance to its application in clinical settings.

## Experimental Models of LET

This section of study refrains from providing an exhaustive review of all previously published models pertaining to hind limb transplantation in rat models and the corresponding immunosuppressive regimens employed therein. We defer such an analysis to the comprehensive review paper authored by Fleissig et al., published in 2020.^45^ Nevertheless, our review aims to comprehensively analyze published limb transplantation models in both small and large animals, with a particular emphasis on assessing the translational applicability of models that have the potential to mimic LET in clinical settings.

### Small Animal Models of Lower Limb Transplantation

#### Hind limb transplantation model in rats

A standard, well-known orthotopic hind limb transplantation model in rats was described by Shapiro et al. in 1978,^39^ which subsequently became the workhorse for VCA studies.^28^ They outlined a replantation model with a proximal amputation of the donor graft. The donor graft amputation below the inguinal ligament and pelvic girdle allowed the inclusion of the anastomosis of the sciatic and femoral nerves, as well as preservation of the profunda branch of the femoral vein. This group was among the first to document any return of functional movement in the grafted limb, although they did not assess sensory response or pain withdrawal. While no movement of the grafted limb was observed, the authors noted good passive movement of the joints at 4 months posttransplant.^39^

While several authors have introduced modifications to this model, the general surgical principles of rat hind limb transplantation have remained unchanged.^33^^,^^34^^,^^41^^,^^45^, ^46^, ^47^, ^48^, ^49^, ^50^ Moreover, isolated parts of the hind limb such as the gastrocnemius muscle^51^ and semimembranous muscle with an epigastric skin flap^18^ have been utilized in transplantation studies. This model has also been adapted for mice. However, the mouse model presents challenges due to the small diameters of femoral vessels and difficulties in vascular anastomosis, necessitating special non-suture microsurgery known as the “cuff” technique. Several groups have used this technique to develop successful orthotopic and heterotopic limb transplant models in mice. Sucher reported success rates of 62% in orthotopic models and 90% in heterotopic models.^40^ Recently, Kern et al.^26^ and Kim et al.^47^ have published an orthotopic forelimb transplantation model.

#### Modifications of rat hind limb transplantation models

##### Rat hindlimb and cremaster allograft transplantation model

This model was created by Ozer et al. in 1999. They harvested the rat hind limb along with the cremaster muscle based on the same vascular pedicle. Their aim was to utilize the cremaster muscle as a window to monitor the microcirculatory changes during allotransplantation settings.^37^^,^^38^ This model enabled the evaluation of leukocyte trafficking during allograft rejection. Throughout the rejection process, the number of rolling and adhering leukocytes and lymphocytes increases due to leukocyte-endothelial interactions.^37^

##### Heterotopic hindlimb allotransplantation in rats

In 2005, Ulusal et al. introduced a nonfunctional heterotopic hind limb allotransplantation model.^41^ The allograft was transplanted to the inguinal region of the recipient. In this model, osteotomy and intramedullary fixation are not needed in the recipient. Unlike the standard orthotopic hind limb model, this model did not require osteotomy and intramedullary fixation in the recipient, resulting in lower morbidity and mortality rates due to reduced operative and ischemia time. Furthermore, problems associated with osteotomy and intramedullary fixation, such as embolism and infection, were not observed. Additionally, the integrity of the femur, bone marrow, and other structures of the lower limb was preserved, providing a more consistent antigenic load compared to the standard hind limb model. Although this model did not yield functional outcomes, it would be more suitable for studies focusing solely on the immunologic aspects of transplantation.^41^

In 2011, Zhang et al.^50^ introduced a nonfunctional heterotopic hind limb allotransplantation model similar to the one previously published by Ulusal et al. in 2005.^41^ Essentially, they suggested a modification wherein the donor whole leg was inset and fixed to the inner aspect of the recipient leg, without bony fixation. This heterotopic model demonstrated a significant reduction in ischemic times and better success rates compared to orthotopic hind limb models, offering a quicker and more successful alternative without compromising the rejection characteristics of the VCA.^41^^,^^49^^,^^50^ This study explored a simplified model of acute limb allograft rejection through the transplantation of the donor hindlimb to the recipient groin area while retaining the original recipient hind limb. However, this model has disadvantages such as friction trauma, as the transplanted limb being dragged, as well as the potential risk to the vascular anastomosis due to shearing trauma.

##### Partial hindlimb tissue transplantation models

Liao et al. introduced a simplified modification of the heterotopic hind limb transplant in 2001. Their model was an osteomyocutaneous partial hindlimb tissue model that incorporated only the disarticulated tibia and fibula, related muscles, and their associated skin. The graft, anchored to the recipient gracilis muscle surface, reduced mass and complexity. In this model, ten vascularized grafts from 5 donor hindlimbs were transplanted contralaterally to syngeneic recipients' inguinal regions. This approach significantly reduced operative time, harvesting time, and warm ischemia time compared to previous whole-limb transplantation models. This model proved to be a suitable composite model for studies on VCA when functional recovery is not the primary focus.^48^

In 2004, Nazzal et al. introduced a less invasive allogeneic partial hindlimb tissue transplantation model, similar to Liao et al.’s previously published model. To minimize the graft size, the limb tissue was transected 5 mm above the knee joint (mid femur) and at the level of the ankle joint, and the gastrocnemius muscle was also excluded. The graft was transplanted subcutaneously in the recipient groin area with externalized donor skin for viability monitoring. This model was less time-consuming and had lower morbidity, providing an opportunity for researchers to explore the immunological hurdles of hindlimb transplantation without the morbidity associated with traditional orthotopic whole-limb transplantation.^49^

In their 2019 study, Fleissig et al.^45^^,^^46^ introduced a new partial hindlimb tissue model similar to those of Liao et al.^48^ and Nazzal et al.’s^49^ previously published models. This model offered a reproducible platform for investigating rejection events and developing therapeutic strategies. The modified surgical model included the tibia and fibula as a bone component via disarticulation from the knee and ankle joints, along with the removal of extraneous muscle and tissue. This modification resulted in reduced stress and pain to the animal, decreased flap mass, and a lower graft profile, contributing to the development of a less invasive VCA model. Specifically, this proposed model enabled serial evaluations through biopsies and imaging, with minimal morbidity. This model held the promise of providing valuable insights into emphasis on VCA rejection mechanisms and vasculopathy.^46^

##### Orthotopic forelimb transplantation model in rodents

Kern et al. introduced a new rat mid-humeral forelimb transplant model to investigate upper extremity functional recovery following transplantation.^26^ In contrast to the standard hind limb model, which is based solely on sciatic nerve repair, they performed median, ulnar, and radial nerve coaptation. In addition to reaching and grasping tasks, they also took advantage of the natural feeding movements of rodents to evaluate forelimb function. Although this model was proposed as an upper limb transplantation model, from the perspective of functional recovery, it can be considered one of the most versatile limb transplantation models for future studies in LET.^26^

##### Pre-transplant modification of the hindlimb allograft

There is a scarcity of literature addressing pre-transplant modification of hind limb allografts to reduce the need for immunosuppression.^23^^,^^30^^,^^31^^,^^35^^,^^36^ For this purpose, the removal of bone marrow from the limb allograft using irradiation and its replacement with recipient bone marrow can prolong allograft survival without the support of any immunosuppressive regimen.^36^ In a rodent study, Lee et al. reported slightly decreased graft rejection following graft irradiation. However, they reported significantly delayed rejection following total body irradiation (TBI).^31^ In a different study, Lee et al. created a chimeric limb allograft (from donor Lewis) via recipient-derived cells (from Brown Norway) and transplanted this chimeric allograft to the Brown Norway (BN) recipient rats. They reported significantly prolonged allograft survival in BN recipients compared to other recipient rat strains (Lewis and Buffalo). This study suggested that when the bone marrow components of the allograft are reconstituted by recipient-derived cells, even though other tissue components of the allograft were allogeneic, prolonged survival of the allograft is possible.^30^

One of the most crucial issues with VCA is graft-versus-host disease (GVHD), even in established chimeras. In chimeric hosts previously created as tolerant to donor antigens, GVHD can be lethal if donor limbs are not manipulated before transplantation.^20^^,^^23^ Gorantla et al. reported that GVHD and destabilization^35^ of chimerism could be eliminated, and rejection-free graft acceptance can be obtained in the chimeric host by irradiating the limb before transplantation.^23^

The manipulation of the immune response to limb allograft by creating the “chimeric limb concept” has also been investigated by Muramatsu et al. The authors designed a protocol for generating a chimeric limb allograft in the rodent model.^35^^,^^36^ First, they irradiated the allograft before transplantation. Then, granulocyte colony-stimulating factor (G-CSF) was administered 4 days after transplantation to activate the migration of the recipient's hematopoietic and immune cells into the allograft bone marrow, under a 28-day immunosuppressive protocol with FK506.^35^ This approach resulted in 1-year allograft survival without rejection. These studies suggest that rapid reconstitution of the donor bone marrow with recipient cells is a crucial factor in determining limb allograft rejection.^30^^,^^31^^,^^35^^,^^36^

However, in the real clinical setting, ethical and clinical issues related to creating a chimeric allograft and recipient, as well as time limitations between the harvesting and transplantation of VCA, impede this kind of manipulation of the allograft. Furthermore, possible radiation-related injuries to the allograft present significant drawbacks in the clinical scenario.^31^

### Large Animal models of lower limb transplantation

Small animal models in rats do not precisely simulate human transplantation from clinical, biological, and immunological aspects (e.g., weight bearing, long distance for nerve regeneration, the large amount of muscle mass). Rats are quadrupeds, making it difficult to employ unilateral hind limb or forelimb transplantation as a preclinical model for LET. Quantitative differences in the gait of bipedal and quadrupedal rats are difficult to distinguish,^52^ despite the fact that bipedalism can be surgically achieved in rats by amputating the forelimb and tail within the first 24 hours of birth.^9^^,^^53^, ^54^, ^55^ Therefore, preclinical large animal models are needed for LET. Some published large animal models of hind limb transplantation exist in swine,^21^^,^^56^ dogs,^22^^,^^29^ and nonhuman primates.^19^ However, most of these models cannot accurately mimic human LET due to problems including, but not limited to, musculoskeletal or partially harvested limb allografts (e.g., distal femur, knee joint, tibia-fibula and surrounding muscles),^25^^,^^32^ and heterotopic hind limb models.^24^^,^^27^ In this article, we only mentioned the closest related large animal model of LET, the porcine orthotopic forelimb VCA model.^21^

#### The porcine orthotopic forelimb VCA model

Fries et al. introduced their orthotopic, load-bearing porcine forelimb VCA model in 2016. Although the authors stated that this model could be used to research different aspects of VCA, including immunologic, bone healing, and nerve and tendon regeneration issues, it can be criticized for their short-term, 14 days follow-up. Furthermore, no immunosuppressive agents were used, and they only observed clinicopathologic signs of rejection.^21^ Therefore, this model can be considered a “pilot study” or a “new large animal model” for future long-term VCA studies.^21^

## Clinical Difficulties of LET

### Challenges related to hemodynamic properties and ischemia-reperfusion injury

The anatomical compartments of the lower extremity differ significantly from those of the upper extremity in terms of volume, muscle mass, and other factors. The surgical team must be especially careful and meticulous in maintaining hemodynamic stability, including resuscitative management, blood pressure, and coagulation markers. Unlike upper extremity transplantation, these cases may require an enormous amount of blood transfusion. Therefore, blood products such as fresh frozen plasma and erythrocyte suspension must be abundantly available before starting the operation. This is crucial because, just after clamp release, a substantial amount of blood will shift into the intravascular compartment, potentially causing fatal events such as severe hypovolemia, ischemia-reperfusion injury (IRI), and electrolyte disproportion. IRI, in particular, can activate innate immunity and increase the risk of allograft rejection.^9^^,^^57^

### Challenges related to nerve regeneration

Nerve regeneration is the sine qua non of the functional success of VCA. Inadequate nerve regeneration leads to delayed muscle function and ultimately results in poor functional outcomes, even in allografts that have survived indefinitely.^58^^,^^59^ In other words, “viable but not functional” allografts mean “functionally graft failure.”

One of the most important reasons for compromised nerve regeneration in LET is the long distance between the nerve coaptation site and the end organs that need to be innervated. Depending on the length of this distance, nerve regeneration may take 1–3 years (with a regeneration speed of 1 mm/day). In the case of LET, excellent physical therapy and rehabilitation may reduce the atrophy of the target muscles that are not being used. However, atrophy due to denervation remains one of the most significant challenges for achieving optimal motor and sensory outcomes.^9^, ^60^

The beneficial effects of FK506 on nerve regeneration and reinnervation of long nerve gaps have been demonstrated in rodent models.^9^^,^^61^, ^62^, ^63^, ^64^, ^65^ However, its usefulness in the clinical VCA setting remains uncertain and is still a topic of speculation. To date, its effectiveness in this context is unknown.^9^

### Challenges related to repetitive trauma during ambulation

The detrimental effects of repeated trauma to the VCA are well-known, especially in hand transplant cases, and this issue is equally significant in LET settings. Unlike the upper extremity, the lower extremity bears the load of the entire body. Therefore, repetitive trauma from shearing forces and cyclic loading on the plantar area and heel, especially in the weight-bearing areas of the foot, may trigger inflammation and rejection, potentially leading to acute rejection episodes in lower extremity allograft. Furthermore, the absence of sensation and proprioception in the plantar area and heel can lead to the development of trophic ulceration and chronic wounds, which also aggravate inflammation and rejection, ultimately resulting in allograft loss.^9^, ^60^, ^66^

### Challenges related to bone fixation and healing

As mentioned above, the lower extremity must be strong enough to bear almost the entire body weight. Therefore, LET requires strong and stable bone fixation. For this purpose, locking plates and screws have been used in most LET patients.^67^ However, some advocate the use of nailing to avoid hardware failure and nonunion problems and to achieve better weight bearing and early mobilization.^68^^,^^69^

Another clinical application of VCA closely related to LET is total knee joint transplantation, which includes femoral and tibial components as well as the knee joint. Diefenbeck et al. performed 6 human vascularized allogeneic knee transplantations. Although all allografts were lost within the first 56 months due to acute or chronic rejection (graft vasculopathy), bone healing was completed within 6 months.^70^, ^71^, ^72^, ^73^ These findings correlated with bone healing observed in hand transplants.^74^, ^75^, ^76^ However, in the long-term follow-up of total knee allografts, stress fractures were reported in 3 out of 6 patients between 14 and 24 months.^72^ Contrarily, bone healing in twin to twin LET transplantation, where immunosuppressive agents were not used, has been reported as satisfactory at the 6th-year posttransplant.^8^ In conclusion, bone healing in VCA may not be adversely affected by immunosuppression in the early period of transplantation. However, its long-term effects on LET remain questionable, and further investigations are needed.

### Controversies in LET

#### Controversies related to indications, patient selection, inclusion, and exclusion criteria

There is still no established indication, patient selection criteria, or inclusion and exclusion criteria for LET. It is unclear whether a below-knee or above-knee stump is more suitable for patient selection.^13^ As anticipated, these debates stem from the scientific community rather than from the patients who would be most affected by the procedure. To address this, Carty et al. and Amy L. Xu et al. performed interesting surveys among lower extremity amputees to understand their attitudes toward LET.^3^^,^^5^ Sixty-seven percent of the respondents stated immunosuppression as a major concern for LET, and nearly 90% of the respondents would change their answers to yes if immunosuppression were not required for transplantation. When considering the reduction of life expectancy, more than one-third of potential candidates found it meaningful if the decrease in life expectancy was between 1 and 4 years, and over 20% of the potential recipients found it meaningful if the reduction was around or over 10 years. In conclusion, despite the risks of lifelong immunosuppression and advancements in lower limb prostheses, a considerable amount of lower limb amputees expressed an interest in seeking LET.^3^^,^^5^

#### Controversies related to cortical plasticity after LET

Following hand transplantation, it has been reported that cortical remodeling occurs and reverses the functional reorganization caused by the amputation.^77^ A 6-year follow-up of the first successful LET demonstrated cortical integration of the limb via functional magnetic resonance imaging (fMRI).^8^ However, this pediatric LET was performed between 3-month-old twins without the use of immunosuppression. There is no long-term survival and follow-up data for adult LET patients, and so cortical integrity data are not available for adult LET patients.^9^

#### Ethical controversies in LET

The development of VCA gives rise to ethical considerations due to decentralized oversight, diverse practices, and a lack of uniformity. The ethical challenge revolves around weighing the functional advantages of limb transplants against the potential risks and adverse effects of immunosuppression. Primary ethical issues include addressing the complex physician-patient relationship, ensuring ethical patient selection, fostering public trust, and establishing standardized success criteria.^15^, ^16^, ^17^

LET introduces ethical complexities, focusing on the equilibrium between advancing well-being and preventing harm in a procedure that enhances life but is not lifesaving.^3^^,^^42^ In contrast to upper extremity VCA,^78^^,^^79^ LET encounters controversy attributable to the latest significant progress in lower extremity prosthetics.^3^

## Discussion

The selection of an appropriate animal model for LET requires consideration of clinically relevant factors, such as nerve regeneration, bone healing, weight-bearing capacity, muscle mass, and perioperative challenges.^80^^,^^81^ Human infants initially walk quadrupedally before developing stable bipedalism,^82^ accompanied by skeletal changes associated with upright posture, like increasing lumbar lordosis, sacral kyphosis, and greater hip and knee extension.^83^ There is no cortical reorganization in infants with the onset of bipedalism.

The literature includes numerous reports of orthotopic limb transplants in quadruped models such as rats,^84^ swine,^21^^,^^56^ dogs,^22^^,^^29^ and nonhuman primates.^19^

Small animal models like rats and mice are predominantly used due to their ease of handling and cost-effectiveness. The orthotopic hindlimb transplantation model in rats, first described by Shapiro et al., is widely used for studying VCA in small animals. However, these models often fall short of mimicking human LET due to differences in muscle mass, weight-bearing requirements, long-distance nerve regeneration challenges, and their innate quadrupedal locomotion behavior (Table 1).

**Table 1:** Animal models of lower extremity transplantation.

Model			Species	Author	The suitability of the properties of the model for translation from animal models to the clinical studies*				
					Nerve regeneration	Bone healing	Weight-bearing	Muscle mass	Perioperative challenges
Small animal models									
Orthotopic hindlimb models	Hindlimb		Mice	Sucher et al.^40^	-	-	-	-	+, -
	Hindlimb		Rat	Shapiro et al.^39^	-	-	-	-	-
	Hindlimb and cremaster		Rat	Ozer et al.^37^^,^^38^^,^^85^^,^^86^	-	-	-	-	-
Heterotopic hindlimb and/or partial hind limb tissue related models	No pretransplant modification	Heterotopic hindlimb allotransplantation	Rat	Ulusal et al.^41^	-	-	-	-	-
		Heterotopic hindlimb allotransplantation	Rat	Zhang et al.^50^	-	-	-	-	-
		Partial hindlimb tissue	Rat	Liao et al.^48^	-	-	-	-	-
		Partial hindlimb tissue	Rat	Nazzal et al.^49^	-	-	-	-	-
		Partial hindlimb tissue	Rat	Fleissig et al.^46^	-	-	-	-	-
	Pre-transplant modification	Graft irradiation, remove donor BM and replace with recipient BM	Rat	Muramatsu et al.^36^	-	-	-	-	-
		Graft irradiation and G-CSF administration under 28 days FK506	Rat	Muramatsu et al.^35^	-	-	-	-	-
		Graft/total body irradiation	Rat	Lee et al.^31^	-	-	-	-	-
		Creation of Chimeric allograft	Rat	Lee et al.^30^	-	-	-	-	-
		Graf irradiation and Chimeric host	Rat	Gorantla et al.^23^	-	-	-	-	-
Orthotopic forelimb models	Forelimb transplantation model		Rat	⁎⁎ Kern et al.^26^	+	-	-	-	-
Large animal models									
Modified hindlimb models	Allograft models include only some part of the hindlimb	Allograft includes distal femur, knee joint, tibia-fibula and surrounding muscles	Swine	Lee et al.^32^	-	-	-	-	-
		Osteomyocutaneous flap includes muscles, knee joint, distal femur, and proximal tibia	Swine	Ibrahim et al.^25^	-	-	-	-	-
		Osteomyocutaneous flap includes muscles, knee joint, distal femur, and proximal tibia	Swine	Hettiaratchy et al.^24^	-	-	-	-	-
	Heterotopic forelimb model	Whole-limb heterotopic autotransplantation model	Swine	Kiermeir et al.^27^	-	-	-	-	-
Orthotopic forelimb model	Forelimb VCA model		Swine	Fries et al.^21^	+, -	+, -	+, -	-	+ / -
Orthotopic forelimb model	Forelimb VCA model		NHP	⁎⁎⁎Stark et al.^87^	+, -	+, -	+, -	-	-

BM: Bone marrow, G-CSF: granulocyte colony-stimulating factor, VCA: vascularized composite allotransplantation, NHP: nonhuman primates

⁎ This section does not show all published properties of the models even if the models have such properties. It only shows that even though the mentioned anatomical repair has been done, and perioperative challenges have been handled in the presented animal model, it can either mimic (+), or not mimic (-) or can partially mimic (+, -) the exact clinical scenario in humans.

⁎⁎ Although this model is proposed as an upper limb transplantation model in rat, from the perspective of functional recovery, it can be considered one of the most versatile small animal limb transplantation models for future studies.

⁎⁎⁎ Although this model is proposed as an upper limb transplantation model, from the perspective of quadrupedal/bipedal locomotion behavior, it can be considered one of the lower limb transplantation models in large animal studies.

Using unilateral hind limb or forelimb transplantation in rats as a preclinical model for LET is problematic because rats are quadrupeds. Although quadrupedal animals like rats and nonhuman primates (e.g., apes) can exhibit occasional bipedal behavior, their walking posture is significantly different from humans, whose skeletal system has evolved specifically for bipedal locomotion, which requires significantly more energy than quadrupedal locomotion.^88^^,^^89^ Bipedalism can be surgically induced in rats by forelimb and tail amputation within 24 hours of birth,^53^, ^54^, ^55^ but quantitative differences in gait between bipedal and quadrupedal rats are hard to distinguish.^52^

Despite the complexity and higher costs associated with large animal models, they provide better translational relevance, especially for functional outcomes like nerve regeneration over long distances and weight-bearing capacity. Large animal models, such as those using swine, offer a more suitable platform for LET research, as they better replicate the anatomical and functional characteristics of human limbs. The orthotopic porcine forelimb VCA model introduced by Fries et al. in 2016 has shown potential in studying immunologic responses, bone healing, and nerve regeneration, although long-term studies are still needed (Table 1 and 2).

**Table 2:** Clinical cases of lower extremity transplantation.

Author	Case	Follow-up	Clinical outcomes
Zuker et al., 2012, Canada^8^^,^^14^	Complete unilateral LET with the hip socket 3-month-old ischiopagus twins one of them with a fetal cardiac anomaly (genetically matched twins)	6 years	Motor and sensory return, ambulation with braces, limb length discrepancy cortical integration of the limb
Cavadas et al., 2011, Spain^7^^,^^43^^,^^90^	First bilateral transfemoral allotransplantation (right mid-and left distal femoral LET)	15 months	Allografts were removed due to primary central nervous system PTLD
Ozkan et al., 2012, Turkey^9^	LET plus bilateral hand transplantation (first triple extremity transplantation)	5 months	Hemodynamic problems in LET (1st post op day LEA removed), Disseminated aspergillosis (5 months after transplantation and patient died)
Nasir et al., 2012, Turkey^10^, ^11^, ^12^	First quadruple extremity transplantation	4 days	The patient died of hemodynamic complications on day 4th after surgery
Arudchelvam et al., 2017, Sri Lanka^4^	Left transfemoral LET	6 years	While the patient is capable of ambulation, his sensory and motor recovery remains incomplete. Additionally, he has developed frequent episodes of lymphedema and lymphangitis in the transplanted limb

PTLD: posttransplant lymphoproliferative disease, LEA: lower extremity allograft, LET: lower extremity transplantation.

Chimpanzees, which share over 99% of human genes, can walk bipedally but without full extension of hip or knee joints. While chimpanzees and other animals use their limbs to support body weight, maintain balance, and produce propulsive force, there is a clear difference between the ischium of chimpanzees and humans. The caudal orientation of the ischium in chimpanzees affects the hamstrings' ability in hip extension, preventing bipedalism, while the dorsal orientation of the ischium in humans is key for bipedalism.^91^ Gait kinematics and electrophysiologic studies in bipedal rats and chimpanzees suggest similarities in neuronal control as observed in humans, at least at the spinal cord level.^55^ This reinforces the inference that observed differences are likely due to skeletal and/or muscular adaptations.

The individual limitations of each model, as discussed, constrain their relevance and value for LET studies. Currently, there is no ideal small or large animal model for LET that allows objective assessment of motor, sensory, or proprioceptive function applicable to human bipedal locomotion.^92^

## Conclusion

To date, the clinical application of LET is confined to only 5 transplant cases worldwide. Nevertheless, the levels of morbidity and mortality are considered quite unacceptable. Numerous unresolved issues, including the challenges of nerve regeneration over long distances, chronic effects of lifelong immunosuppression, and LET-specific perioperative and patient-related complications, hinder its routine clinical application. Addressing these concerns could make LET a more viable reconstructive option for carefully selected recipients in the future.

## References

[1] Dubernard J.M. (1999). [The first transplantation of a hand in humans. Early results]. Chirurgie.

[2] Devauchelle B. (2006). First human face allograft: early report. Lancet.

[3] Xu A.L., Humbyrd C.J. (2022). Ethical challenges in vascularized composite allotransplantation of the lower extremity: lessons learned from hand transplantation and implications for the future. Plast Aesthet Res.

[4] Arudchelvam J. (2018). Trans-femoral lower limb transplantation in a Sri Lankan patient: a case report and surgical technique. Ceylon Med J.

[5] Carty M.J. (2014). Attitudes regarding lower extremity allotransplantation among lower extremity amputees. Plast Reconstr Surg.

[6] Carty M.J. (2013). The case for lower extremity allotransplantation. Plast Reconstr Surg.

[7] Cavadas P.C. (2013). Bilateral transfemoral lower extremity transplantation: result at 1 year. Am J Transplant.

[8] Fattah A. (2011). The first successful lower extremity transplantation: 6-year follow-up and implications for cortical plasticity. Am J Transplant.

[9] Gorantla V.S., Tepe V., Peterson C. (2017). Full Stride.

[10] Landin L., Dominguez A., Sanchez-Sanchez M. (2014). Discussion of lessons learned from the first quadruple extremity transplantation in the world: the logic of massive allograft transplantation. Ann Plast Surg.

[11] Nasir S. (2014). Lessons learned from the first quadruple extremity transplantation in the world. Ann Plast Surg.

[12] Swanson E.W., Brandacher G., Gordon C.R. (2014). Discussion of lessons learned from the first quadruple extremity transplantation in the world: comments and concerns regarding quadruple extremity allotransplantation. Ann Plast Surg.

[13] Swanson E.W. (2015). Lower extremity allotransplantation: are we ready for prime time?. Vasc Compos Allotransplantation.

[14] Zuker R.M. (2006). First successful lower-extremity transplantation: technique and functional result. J Reconstr Microsurg.

[15] Benatar D., Hudson D.A. (2002). A tale of two novel transplants not done: the ethics of limb allografts. BMJ.

[16] Caplan A.L. (2019). Emerging ethical challenges raised by the evolution of vascularized composite allotransplantation. Transplantation.

[17] Kumnig M. (2022). Psychosocial and bioethical challenges and developments for the future of vascularized composite allotransplantation: a scoping review and viewpoint of recent developments and clinical experiences in the field of vascularized composite allotransplantation. Front Psychol.

[18] Agaoglu G., Siemionow M. (2005). Combined semimembranosus muscle and epigastric skin flap: a new model of composite-free flap in the rat. Ann Plast Surg.

[19] Cendales L.C. (2005). Composite tissue allotransplantation: development of a preclinical model in nonhuman primates. Transplantation.

[20] Foster R.D. (2001). Mixed allogeneic chimerism as a reliable model for composite tissue allograft tolerance induction across major and minor histocompatibility barriers. Transplantation.

[21] Fries C.A. (2016). A porcine orthotopic forelimb vascularized composite allotransplantation model: technical considerations and translational implications. Plast Reconstr Surg.

[22] Goldwyn R.M. (1966). Canine limb homotransplantation. Plast Reconstr Surg.

[23] Gorantla V.S. (2003). Composite tissue allotransplantation in chimeric hosts: part I. Prevention of graft-versus-host disease. Transplantation.

[24] Hettiaratchy S. (2004). Tolerance to composite tissue allografts across a major histocompatibility barrier in miniature swine. Transplantation.

[25] Ibrahim Z. (2013). A modified heterotopic swine hind limb transplant model for translational vascularized composite allotransplantation (VCA) research. J Vis Exp.

[26] Kern B. (2017). A novel rodent orthotopic forelimb transplantation model that allows for reliable assessment of functional recovery resulting from nerve regeneration. Am J Transplant.

[27] Kiermeir D.M. (2013). Evaluation of a porcine whole-limb heterotopic autotransplantation model. Microsurgery.

[28] Kim S.K. (1984). Use of cyclosporin A in allotransplantation of rat limbs. Ann Plast Surg.

[29] Lance E.M. (1971). Transplantation of the canine hind limb. Surgical technique and methods of immunosuppression for allotransplantation. A preliminary report. J Bone Joint Surg Am.

[30] Lee W.P. (2001). Donor modification leads to prolonged survival of limb allografts. Plast Reconstr Surg.

[31] Lee W.P. (1995). Prolonged survival of vascularized limb tissue allografts by donor irradiation. J Surg Res.

[32] Lee W.P. (2001). Tolerance to limb tissue allografts between swine matched for major histocompatibility complex antigens. Plast Reconstr Surg.

[33] Lipson R.A. (1983). Vascularized limb transplantation in the rat. I. Results with syngeneic grafts. Transplantation.

[34] Lipson R.A. (1983). Vascularized limb transplantation in the rat. II. Results with allogeneic grafts. Transplantation.

[35] Muramatsu K. (2010). Myeloablative irradiation and granulocyte colony-stimulating factor prolong the survival of chimeric limb allografts in a rodent model. Plast Reconstr Surg.

[36] Muramatsu K. (2012). Immunomodulatory effects of pre-irradiated extremity allograft in the rodent model. J Plast Reconstr Aesthet Surg.

[37] Ozer K. (1999). In vivo microscopic assessment of cremasteric microcirculation during hindlimb allograft rejection in rats. Plast Reconstr Surg.

[38] Ozer K., Siemionow M. (2001). Combination of anti-ICAM-1 and anti-LFA-1 monoclonal antibody therapy prolongs allograft survival in rat hind-limb transplants. J Reconstr Microsurg.

[39] Shapiro R.I., Cerra F.B. (1978). A model for reimplantation and transplantation of a complex organ: the rat hind limb. J Surg Res.

[40] Sucher R. (2010). Mouse hind limb transplantation: a new composite tissue allotransplantation model using nonsuture supermicrosurgery. Transplantation.

[41] Ulusal A.E. (2005). Heterotopic hindlimb allotransplantation in rats: an alternative model for immunological research in composite-tissue allotransplantation. Microsurgery.

[42] Kodali N.A. (2024). A world update of progress in lower extremity transplantation: what's hot and what's not. Ann Plast Surg.

[43] Cavadas P.C. (2015). Primary central nervous system posttransplant lymphoproliferative disease in a bilateral transfemoral lower extremity transplantation recipient. Am J Transplant.

[44] Janarthanan, R., *Personal communication with Dr. Arudchelvam* January 17, 2024.

[45] Fleissig Y.Y. (2020). Evolution of the rat hind limb transplant as an experimental model of vascularized composite allotransplantation: Approaches and advantages. SAGE Open Med.

[46] Fleissig Y. (2019). Modified heterotopic hindlimb osteomyocutaneous flap model in the rat for translational vascularized composite allotransplantation research. J Vis Exp.

[47] Kim M. (2018). Improved cuff technique and intraoperative detection of vascular complications for hind limb transplantation in mice. Transplant Direct.

[48] Liao T.C. (2001). An osteomyocutaneous transplantation model on the rat. Microsurgery.

[49] Nazzal J.A. (2004). Heterotopic limb allotransplantation model to study skin rejection in the rat. Microsurgery.

[50] Zhang Z. (2011). A modified rat model of acute limb allograft rejection. Transplant Proc.

[51] Tan C.M. (1991). Vascularized muscle allografts and the role of cyclosporine. Plast Reconstr Surg.

[52] Bailey A.S. (2001). A comparison between bipedal and quadrupedal rats: do bipedal rats actually assume an upright posture?. Spine.

[53] Goff C.W., Landmesser W. (1957). Bipedal rats and mice; laboratory animals for orthopaedic research. J Bone Joint Surg Am.

[54] Kay E.D., Condon K. (1987). Skeletal changes in the hindlimbs of bipedal rats. Anat Rec.

[55] Moravec S.J., Cleall J.F. (1987). An assessment of posture in bipedal rats. Am J Anat.

[56] Fries C.A., Tuder D.W., Davis M.R. (2015). Preclinical models in vascularized composite allotransplantation. Curr Transplant Rep.

[57] Khalil A.A., Aziz F.A., Hall J.C. (2006). Reperfusion injury. Plast Reconstr Surg.

[58] Faroni A. (2015). Peripheral nerve regeneration: experimental strategies and future perspectives. Adv Drug Deliv Rev.

[59] Midha R., Binder M.D., Hirokawa N. (2009). Encyclopedia of neuroscience.

[60] Kulahci Y., Karagoz H., Zor F. (2018). Experimental Models of Penile and Lower Limb Transplantation: Are They Really Translational?. Curr Transplant Rep.

[61] Navarro X. (2001). Effects of FK506 on nerve regeneration and reinnervation after graft or tube repair of long nerve gaps. Muscle Nerve.

[62] Sobol J.B. (2003). Effects of delaying FK506 administration on neuroregeneration in a rodent model. J Reconstr Microsurg.

[63] Udina E. (2003). FK506 enhances reinnervation by regeneration and by collateral sprouting of peripheral nerve fibers. Exp Neurol.

[64] Udina E. (2002). Bimodal dose-dependence of FK506 on the rate of axonal regeneration in mouse peripheral nerve. Muscle Nerve.

[65] Yang R.K. (2003). Dose-dependent effects of FK506 on neuroregeneration in a rat model. Plast Reconstr Surg.

[66] Hautz T. (2012). Mechanisms and mediators of inflammation: potential models for skin rejection and targeted therapy in vascularized composite allotransplantation. Clin Dev Immunol.

[67] Egol K.A. (2004). Biomechanics of locked plates and screws. J Orthop Trauma.

[68] Brumback R.J. (1999). Immediate weight-bearing after treatment of a comminuted fracture of the femoral shaft with a statically locked intramedullary nail. J Bone Joint Surg Am.

[69] Wu C.C., Tai C.L. (2006). A biomechanical comparison of unlocked or locked reamed intramedullary nails in the treatment of mid-third simple transverse femoral shaft fractures. Chang Gung Med J.

[70] Diefenbeck M. (2011). Allograft vasculopathy after allogeneic vascularized knee transplantation. Transpl Int.

[71] Diefenbeck M. (2006). Management of acute rejection 2 years after allogeneic vascularized knee joint transplantation. Transpl Int.

[72] Diefenbeck M. (2007). Outcome of allogeneic vascularized knee transplants. Transpl Int.

[73] Hofmann G., Lanzetta M., Dubernard J. (2007). Hand transplantation.

[74] Cavadas P.C. (2011). Bone healing after secondary surgery on hand allografts under sirolimus-based maintenance immunosuppression. Ann Plast Surg.

[75] Gabl M., Lanzetta M., Dubernard J. (2007). Hand Transplantation.

[76] Gabl M. (2004). Bilateral hand transplantation: bone healing under immunosuppression with tacrolimus, mycophenolate mofetil, and prednisolone. J Hand Surg Am.

[77] Dubernard J.M. (2013). Fifteen years later: main lessons from composite tissue allografts. Clin Transpl.

[78] Bertrand A.A. (2014). Changing attitudes toward hand allotransplantation among North American hand surgeons. Ann Plast Surg.

[79] Mathes D.W. (2009). A survey of North American hand surgeons on their current attitudes toward hand transplantation. J Hand Surg Am.

[80] Schmitt D. (2003). Insights into the evolution of human bipedalism from experimental studies of humans and other primates. J Exp Biol.

[81] Ambrose S.H. (2001). Paleolithic technology and human evolution. Science.

[82] Roberts A., Thorpe S. (2014). Challenges to human uniqueness: bipedalism, birth and brains. J Zool.

[83] Gruss L.T., Schmitt D. (2015). The evolution of the human pelvis: changing adaptations to bipedalism, obstetrics and thermoregulation. Philos Trans R Soc Lond B Biol Sci.

[84] Perez-Abadia G. (2003). Low-dose immunosuppression in a rat hind-limb transplantation model. Transpl Int.

[85] Siemionow M. (2000). Microcirculatory window for early detection of allograft rejection. Ann Plast Surg.

[86] Ozer K., Siemionow M.Z. (2015). Plastic and Reconstructive Surgery.

[87] Stark G.B. (1987). Hand transplantation in baboons. Transplant Proc.

[88] Hosoido T. (2013). Qualitative comparison between rats and humans in quadrupedal and bipedal locomotion. J Behav Brain Sci.

[89] Steudel K. (1996). Limb morphology, bipedal gait, and the energetics of hominid locomotion. Am J Phys Anthropol.

[90] de Lago M. (2011). World's first double leg transplantation is carried out in Spain. BMJ.

[91] Shapiro L.J., Anapol F.C., Jungers W.L. (1997). Interlimb coordination, gait, and neural control of quadrupedalism in chimpanzees. Am J Phys Anthropol.

[92] Alexander R.M. (2004). Bipedal animals, and their differences from humans. J Anat.

